# Extradural Anesthesia in a Case of Mild Head Injury

**DOI:** 10.7759/cureus.16475

**Published:** 2021-07-19

**Authors:** Ankita Kabi, Shipra Tandon, Priya T Kandy

**Affiliations:** 1 Anesthesiology, All India Institute of Medical Sciences, Rishikesh, IND; 2 Emergency Medicine, All India Institute of Medical Sciences, Rishikesh, IND

**Keywords:** polytrauma, epidural anaesthesia, mild head injury, intracranial pressure, pain management

## Abstract

We experienced a case posted for bilateral lower limb surgery in a patient having mild traumatic brain injury (TBI), where administration of graded epidural anesthesia led to agitation, probably resulting from the transient elevation of intracranial pressure (ICP). Due to the wide range of benefits provided by regional anesthesia, an anesthetist should be aware of the possible options for perioperative management to best handle such polytrauma cases. In this case, agitation was managed with a bolus of benzodiazepine and maintenance infusion of dexmedetomidine while the six-hour-long surgery continued with epidural anesthesia. This patient was a smoker who had bronchospasm and underlying pneumonia precluding a high risk for postoperative ventilatory support if only general anesthesia was administered. Post-surgery the patient was conscious, oriented, and pain-free leading to early mobilization and discharge from the hospital. The patient did not report any neurological deterioration in a follow-up period of one month.

## Introduction

Anesthesia consideration in patients with head injury posted for extracranial surgeries should include neuroprotection to avoid secondary brain injury. Regional anesthesia in such patients offers excellent pain control but is still debatable. The risk of inadvertent dural puncture while administering epidural anesthesia can be detrimental in such patients. Few animal [1] and human [2] studies have shown a transient increase in intracranial pressure (ICP) following a large volume bolus of extradural injection of local anesthetic (LA) or saline [3]. On the other hand, the benefits of regional anesthesia over general anesthesia are many namely superior analgesia, reduced opioid consumption, reduced pulmonary complications, improved gastrointestinal function, early mobilization, reduced need of thrombo-prophylaxis, early discharge, and higher patient satisfaction [4]. There are some reports about the safety and efficacy of epidural anesthesia in obstetric practice with increased ICP [5]. Here we report a case of the use of epidural anesthesia in a polytrauma patient with mild traumatic brain injury (TBI).

## Case presentation

**Figure 1:**
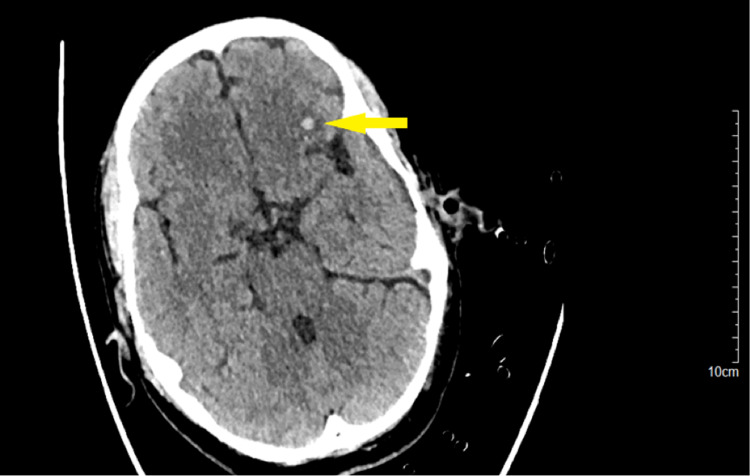
Non-contrast CT scan of the patient showing contusion in the left frontal lobe.

**Figure 2:**
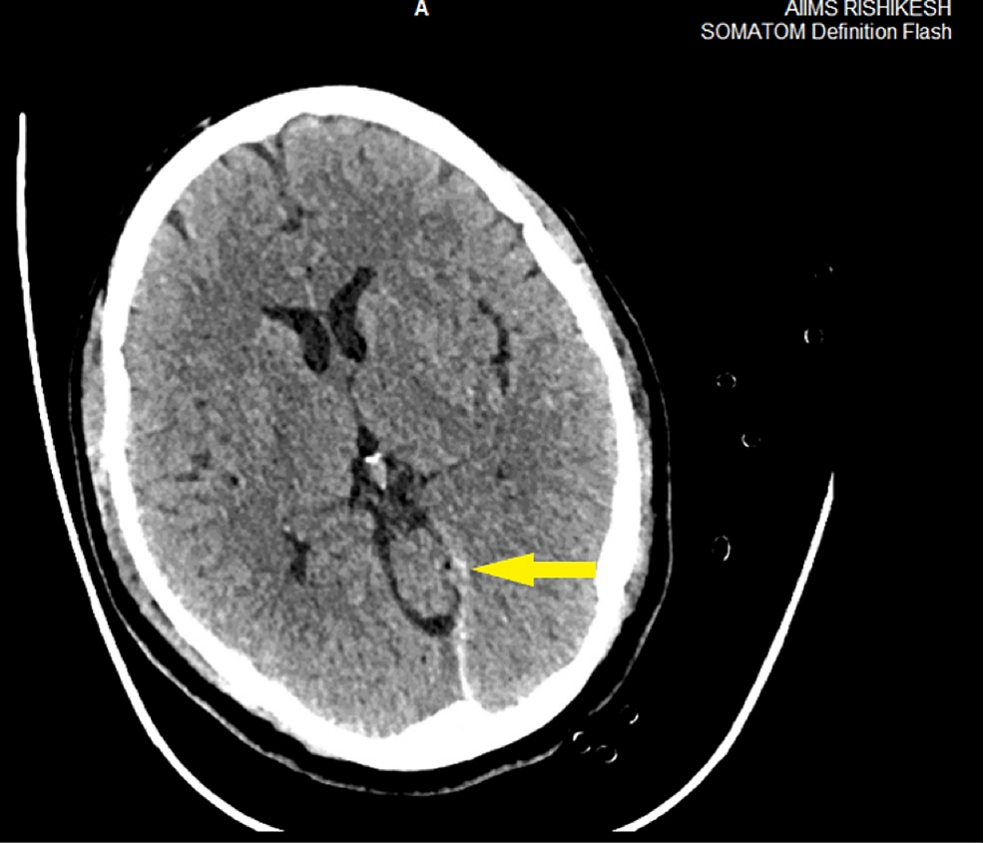
Non-contrast CT scan of the patient showing subarachnoid hemorrhage in the left Sylvian fissure.

A 51-year-old male, with a history of occasional smoking and alcohol intake, sustained a fracture of tibia and fibula in bilateral lower limbs and left frontal lobe contusion (Figure 1) with left Sylvian fissure subarachnoid hemorrhage (Figure 2) after a road traffic accident.

 

On the measurement of optic nerve sheath diameter (ONSD) of the patient using a non-contrast computed tomography (NCCT) scan of the head, it was found that the right side was 7.77 mm while the left side was 7.41 mm. He was posted for closed reduction and intra-locking nailing of the left and right tibia along with open reduction and plating of the right fibula. On pre-anesthetic check-up, his chest auscultation revealed rhonchi in all the lung fields and coarse crepitations in the right inframammary area. He was prescribed nebulization with ipratropium, budesonide, and a course of amoxicillin clavulanate for suspected community-acquired pneumonia. After neurosurgery consultation, prophylactic antiepileptic therapy in the form of phenytoin was also initiated in view of mild head injury. After discussion with the surgeon, the patient was posted for surgery after completion of antibiotics and improvement in chest condition. His non-ambulatory status due to bilateral fractures posed him a greater risk of pulmonary complications. On further discussion with the surgeon, the patient was posted within seven days of his admission The provisional fitness was given under ASA PS-III.

 On the day of surgery, the patient was conscious and co-operative with Glasgow Coma Scale (GCS) -- 15/15. Standard monitors -- five lead electrocardiogram (ECG), non-invasive blood pressure (NIBP), and pulse oximeter were attached. Two wide bore IV cannulas were secured in both arms. The plan for anesthesia was decided to be regional considering his non-optimized pulmonary condition. The requisite informed consent was obtained. Under all aseptic precautions, epidural anesthesia was given at L1-L2 interspace in sitting position via midline approach. Loss of resistance was identified at a depth of 5 cm and an epidural catheter was fixed at 11 cm from the skin.

 Some 3 mL of 1.5% lignocaine with adrenaline was given as a test dose to check for signs of intrathecal or intravascular cannulation. A total volume of 10 mL lignocaine was given in a graded fashion over 30 min and epidural infusion of bupivacaine 0.5% started at 5 mL/h. The sensory and motor blockade at the thoracic dermatomal level (T10) was attained bilaterally and surgery started. Around 15-20 min later, the patient became agitated and started removing chest leads. Immediately the infusion was paused. The vitals were stable, hence a bolus of midazolam 2 mg IV was given. Loading dose of dexmedetomidine was initiated at a dose of 1 mcg/kg/h IV over 10 min followed by a maintenance dose of 0.5 mcg/kg/h IV. Ultrasonography was not available for measuring the ONSD at the time of agitation. After achieving a Ramsay sedation score of 2-3, the epidural infusion was initiated again.

 Intraoperatively, hemodynamics was maintained well with a mean arterial pressure > 65 mmHg throughout, blood glucose was 141 mg/dL, arterial blood gas (ABG) pH - 7.43, pCO2 - 29, pO2 - 155, HCO3 - 19.7, Na+ - 138, K+ - 4.2, and Hb - 8.8. The surgery lasted for six hours during which one unit of packed red blood cells was transfused due to ongoing blood loss. The GCS was 15 after completion of surgery. There were no signs of focal neurological deficits. Post-operative NCCT head showed no new changes and anti-epileptics were advised to be continued. The ONSD measured was 7.12 mm on the right side and 6.54 mm on the left side signifying no continued increase in ICP due to extradural anesthesia. In the ward, the patient was comfortable, pain-free and could mobilize his bilateral lower limbs. His epidural catheter was removed the next day and he was started IV analgesics namely paracetamol and tramadol later converted to oral formulation. He was discharged on post-operative day 3 of the surgery. On follow-up via telephone after one month, the patient did not report any features of deterioration in neurological status.

## Discussion

There has been a significant increase in trauma cases as a consequence of rapid urbanization. In a retrospective study conducted by Abcejo, it was reported that 552 patients had surgery within one week after concussive injury of which 29 (5%) had surgical procedures unrelated to their concussion-producing traumatic injury. Orthopedic and general surgical procedures accounted for 57% of these procedures [6]. Mostly, general anesthesia (GA) is preferred in such patients. With certain limitations of GA, a regional technique may be used. In these patients with decreased intracranial compliance, epidural anesthesia should be used with extreme caution [7]. Any amount of volume injected in this closed space extending from foramen magnum till S2, will displace a similar amount of blood and cerebrospinal fluid (CSF), as per the Monroe Killie Doctrine which was first published by Buchchloz and Lesse in 1951 [8]. The increased pressure due to extradural injection is transmitted to the dural sac, which then leads to CSF shift and increases in ICP in accordance with the intracranial compliance. This direct correlation of increase in ICP following an extradural injection, with the onset of symptoms, was shown by Green [9]. Standard epidural injections of 5 or 10 mL of anesthetic solutions (or 0.9% saline) produced a substantial rise in ICP in two patients who had suffered a head injury more than a week previously, explicable as the effect of compression of the dural sac shifting CSF back into the intracranial compartment. The clinical significance of this increase has been questioned, but slow injection of incremental volumes of local anesthetic has been recommended [10]. Paul and Wildsmith [3] demonstrated that larger volumes result in greater pressure at the end of injection but were not sustainable beyond one minute.

Zabolotskikh and Trembach demonstrated a non-invasive method to determine ICP in 65 patients by using opthalmodynamometry wherein they concluded that a continuous epidural infusion with a low concentration of local anesthetic appears to be safe in patients with increased ICP [11]. The main limitation of this method is that it estimates only the trend of ICP while the abrupt changes may remain unnoticed.

The safety of epidural anesthesia in cases with mild TBI is questionable and controversial. Yet, there are few reports of usage of the same for successful management in obstetric practice [4]. If the benefits outweigh the risks involved, close monitoring of GCS, ONSD can be done intraoperatively. The anesthesiologist must counsel the patient and relatives explaining both the merits and demerits of the technique of anesthesia and obtain informed consent for the conversion of anesthesia to general if the need arises. The neuroprotective action of dexmeditomidine is well known making it an excellent sedative agent for such cases.

## Conclusions

The patient experienced agitation which probably can be attributed to an increase in ICP following extradural injection of a bolus of 10 mL of lignocaine. But it was a transient single episode. The continuous epidural infusion at a low rate did not bring any immediate change in the ICP, as the GCS post-operatively was full as well as on follow-up after a month. Due to effective analgesia, he could be mobilized early hence decreasing his length of hospital stay. Thus, trauma anesthesiologists should be familiar with the benefits and risks of all the anesthetic modalities and manage in the best interest of the patients.
